# Analysis of US Marketed Artemisinin Supplements for Use in Dogs

**DOI:** 10.1111/jvp.13480

**Published:** 2024-08-24

**Authors:** Alyssa R. Berman, Adam J. Birkenheuer, Emily L. Sorah, Mark G. Papich

**Affiliations:** ^1^ Department of Clinical Sciences, College of Veterinary Medicine North Carolina State University Raleigh North Carolina USA; ^2^ Veterinary Hospital Pharmacy, College of Veterinary Medicine North Carolina State University Raleigh North Carolina USA; ^3^ Department of Molecular Biomedical Sciences, College of Veterinary Medicine North Carolina State University Raleigh North Carolina USA

**Keywords:** Artemisinin, *Babesia*, dogs, HPLC, supplement

## Abstract

Oral artemisinin has antiparasitic activity and may help improve treatment success rates in dogs infected with *Babesia gibsoni*. However, these artemisinin products are unapproved and unregulated botanical supplements. They have not been evaluated for safety and efficacy or for strength, purity, or quality compared with a reference standard. Before considering these products for a clinical study, we evaluated the strength of four suppliers of artemisinin capsules using an high‐performance liquid chromatography method validated in our laboratory. We found that the four artemisinin‐labeled products that were tested had high within product and between product variability in capsule strength compared with the stated capsule strength on the product label. No products met the acceptance criteria of the United States Pharmacopeia and International Council for Harmonisation (ICH) as well as the criteria adapted by the authors. One product had no detectable artemisinin, and the other three products were much higher than the stated label strength. The results of this study reinforce the importance of testing unapproved and unregulated supplements before recommending a supplement for clinical use in dogs.

Artemisinin (m.w. 282.33 Da; C_15_H_22_O_5_; synonyms include Qinghaosu, 63,968‐64‐9, Artemisinine) is a botanical supplement that is a sesquiterpene lactone and is derived from *Artemisia annua* (sweet wormwood plant) (National Center for Biotechnology Information 2024). In humans, artemisinin derivatives have been widely used for treatment of malaria (Bridgford et al. 2018; Krishna et al. 2008; Yang et al. 2020). They have also been shown to exhibit other antiparasitic, antibacterial, antifungal, anti‐inflammatory, antitumor, and antioxidant activity (Kim et al. 2015; Wang et al. 2018; Krishna et al. 2008; Li et al. 2020; Shi et al. 2015). Artemisinin compounds and derivatives are endoperoxide‐containing natural products that have shown anticancer activity in dogs and cats with tumors (Rutteman et al. 2013; Saeed et al. 2020), with higher survival times when given in addition to standard treatment (Saeed et al. 2020), and potential prolonged survival times when given following surgical tumor removal (Breuer and Efferth 2014). Artemisinin is also being considered for antiparasitic use in veterinary species both experimentally and anecdotally. When used for treatment of leishmaniosis, artemisinin and its derivatives exhibited improvement in clinical signs in a cat (Hopke et al. 2021) and higher treatment efficacy in dogs compared with the current first‐line treatment (Medkour et al. 2020). In vitro evaluations of artemisinin compounds and derivatives have shown promising inhibitory effects against *Babesia gibsoni* isolated from dogs (Goo et al. 2010; Iguchi et al. 2015; Matsuu et al. 2008). The combination of artesunate (an artemisinin derivative) with atovaquone and azithromycin has produced favorable results for treatment of dogs with *Babesia gibsoni* in Slovakia, with a complete elimination of parasitemia in all 12 cases (Karasová et al. 2022). The artesunate, used by Karasova et al., was a chemical grade compound and is not approved for use in the United States. While intravenous artesunate is approved by the Food and Drug Administration (FDA) for treating malaria in humans, it is not readily available for use in veterinary patients in the United States. Therefore, future studies are warranted and ongoing clinical trials are planned by our group to evaluate the efficacy of artemisinin oral supplements as an alternative to artesunate for treatment of *Babesia gibsoni* in dogs. Our aim is to determine if adding artemisinin as an adjunct to standard treatment with azithromycin and atovaquone will improve treatment outcomes in *Babesia gibsoni* infected dogs. We are aware that artemisinin supplements are currently being used in a combined treatment protocol for babesiosis in dogs (Birkenheuer personal communication).

However, artemisinin oral supplements, like all dietary supplements, are not FDA approved. As a result, the quality, purity, and strength of these supplements are variable. There is no United States Pharmacopeia (USP) monograph for the artemisinin botanical supplements available commercially through retail markets. Therefore, it is essential to analyze artemisinin strength from these products to ensure quality assurance. An efficacy study to examine *Babesia gibsoni* treatment in dogs cannot be performed without understanding the quality of these products.

Our objective was to develop a high‐performance liquid chromatography (HPLC) assay for artemisinin following guidelines from another study (Lapkin et al. 2009). Secondly, with assistance from our pharmacy, we selected four popular brands of artemisinin capsules available from commercial sources (described in Table 1 note) and evaluated their strength compared with a pure analytical reference standard. There are no acceptance criteria for oral artemisinin products because a USP monograph does not exist. Therefore, we adopted the widely accepted USP criteria for evaluating unapproved products, which is a range within ± 10% (i.e., 90%–110%) of the stated label strength (USP Compounding Expert Committee et al. 2014).

**TABLE 1:** Manufacturer (source), capsule size, and average strength from six replicates of four sources of artemisinin.

Laboratory label	Manufacturer	Capsule size (mg)	Average percent strength of six capsules	Standard deviation
A	HumanX	250	0.00	0.00
B	Researched Nutritionals	125	160.98	25.39
C	Allergy Research Group	100	181.49	39.85
D	Hepalin.com	100	153.58	33.99

*Note*: Source, address, and other information for each product used in the study. HumanX LLC, Artemisinin. 302 Washington St. #151, San Diego, CA 92103. https://www.shophumanx.com/products/artemisinin
Researched Nutritionals, Artemisinin Solo. 281 Pamela Way Suite 101, Buellton, CA 93427. https://www.researchednutritionals.com/product/artemisinin‐solo/
Allergy Research Group, Artemisinin. 2300 South Main Street, South Salt Lake, UT 84115. https://www.allergyresearchgroup.com/artemisinin/
Artemix LLC, Hepalin 100. P.O. Box 2858, Palos Verdes, CA 90274. https://www.hepalin.com/hepalin100.htm/.

An analytical reference solution was obtained by dissolving a pure analytical reference standard of artemisinin (USP, www.USP.org) in methanol to a concentration of 1 mg/mL. This solution was stable in the refrigerator, protected from light, for at least 2 weeks. Additional dilutions were performed with a solution of 40% acetonitrile and 60% ultrapure water. The calibration samples consisted of five standards, plus a blank, ranging from 10 to 200 μg/mL. These fortified samples were analyzed along with the incurred samples using the procedure described below.

Six randomly chosen capsules from each formulation were selected for analysis. Each capsule was opened and dissolved in a volume of methanol equivalent to the nominal strength of the capsule. The solution was thoroughly mixed in a beaker using a magnetic stir bar. An aliquot of this solution was diluted with a solution of 40% acetonitrile and 60% water to a nominal concentration of 50 or 100 μg/mL, depending on the label strength of the capsule. Each sample was loaded into a Whatman Mini‐UniPrep syringeless filter device, pore size 0.2 μm (Part # US503NPEAQU) to remove any undissolved excipients. The sample vials were loaded into the HPLC autosampler.

The HPLC system (Agilent 1200 series system, Agilent Technologies, Wilmington, DE) consisted of a quaternary solvent delivery system, autosampler, and ultraviolet (UV) detector. The data were collected with chromatogram integration software (Agilent Technologies OpenLAB CDS software, Agilent Technologies, Wilmington, DE).

Ten μL of the prepared sample was injected into the HPLC system, with a mobile phase consisting of 30% pure water, 20% methanol, and 50% acetonitrile at a flow rate of 1 mL/min. The analytical column was a reverse‐phase Zorbax Eclipse XDB C8 4.6 × 15, 5 μm column at 40°C (Zorbax, Agilent technologies, Wilmington DE). The wavelength of detection was 214 nm. The peak of interest eluted at approximately 3.6 min.

Our acceptance criteria for the assay were based on a linear calibration curve with a R^2^ value of at least 0.99, and QC samples that were within 15% of the nominal concentration. The limit of quantification (LOQ) and limit of detection (LOD) were based on the lowest value on a linear calibration curve and acceptable signal‐to‐nose ratio as described in other guidelines (ICH Q2 (R1) 2005; United States Pharmacopeia 2023).

The assay met our acceptance criteria, and all detected peaks were above the LOQ for the assay. The results of the analysis for four products are provided in Table 1 and Figure 1. Representative chromatograms from the assay are shown in Figure 2.

**FIGURE 1:**
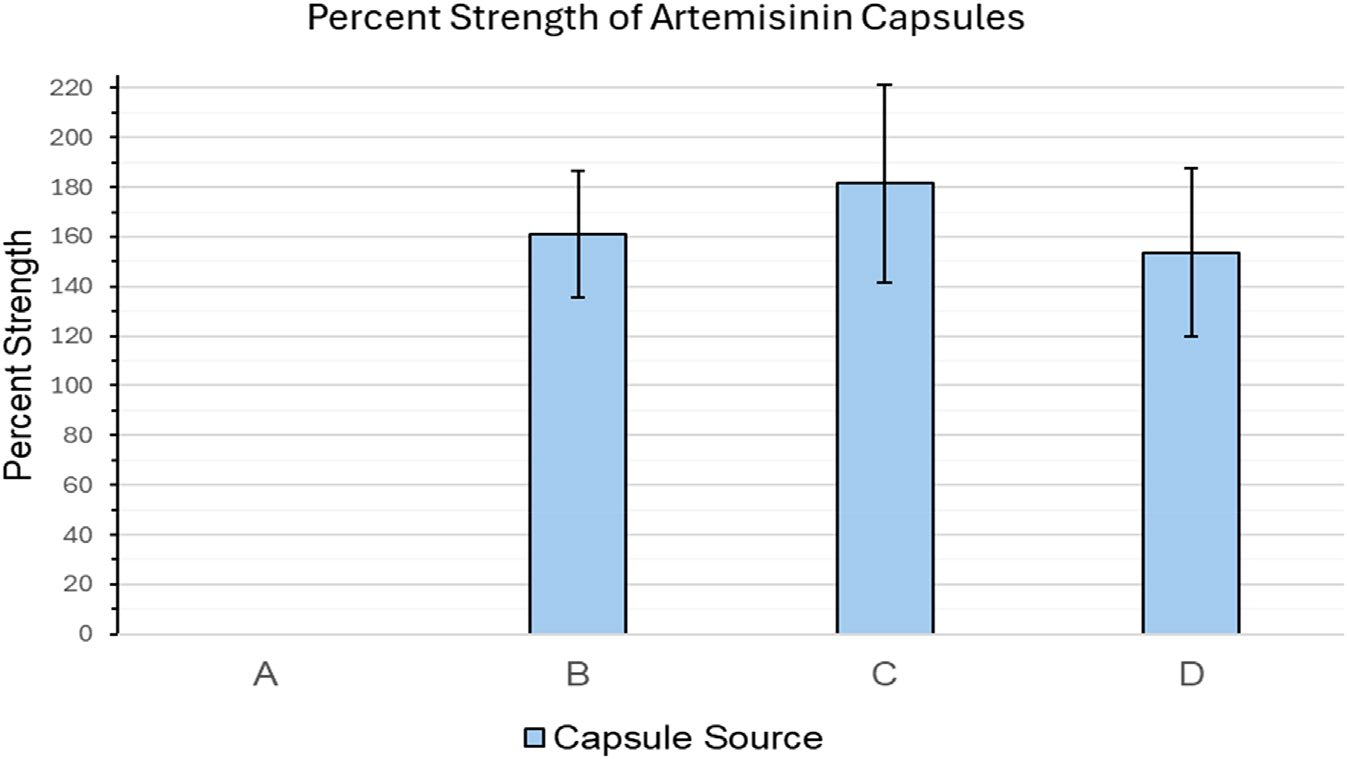
Strength of six replicates artemisinin capsules evaluated by HPLC. Refer to Table 1 for the source of each product. Each bar represents the mean, with error bars representing ± one standard deviation.

**FIGURE 2:**
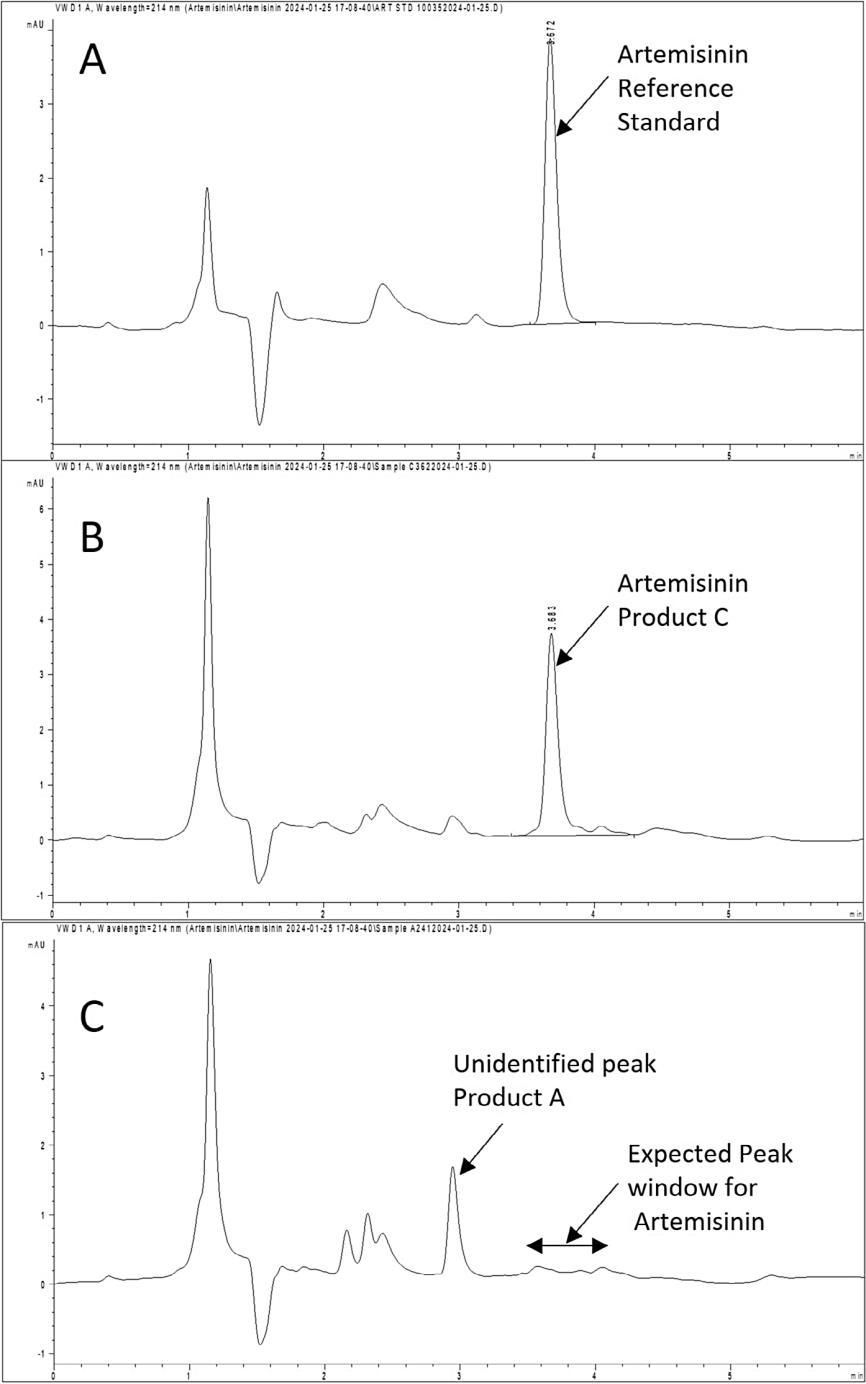
Representative Chromatograms. Panel A shows the chromatogram for artemisinin reference standard with a peak eluting at approximately 3.6 min. Panel B shows a representative chromatogram for a peak of one of the tested products (Product C). Panel C shows a representative chromatogram for Product A showing no detectable artemisinin eluting in the window for artemisinin, but an unidentified peak eluting earlier.

Table 1 and Figure 1 show a high variability between and among products analyzed. One source (Product A in the table and figure) had no detectable artemisinin. The other three sources varied widely, and all exceeded the labeled strength. No products had an average strength that met our acceptance criteria of range of 90%–110% of the labeled strength.

We observed a large additional peak in the chromatograph that eluted before the window of detection for artemisinin for Product A (Figure 2). This additional peak could potentially be caused by contaminants or an artemisinin derivative; however, without further study and analysis (e.g., mass spectrometry), we cannot identify the source of this peak.

According to USP criteria for unapproved formulations, the acceptable range of strength of a formulation should be within ± 10% of the nominal strength on the label (www.USP.org). However, out of the four artemisinin products tested, none were within this acceptable range of strength. This deviation from labeled strength among products highlights the importance of quality control studies for products intended for clinical use in dogs.

Compared with the labeled strength of each capsule, there was large variability in artemisinin concentrations found within and between products analyzed for this study. This variability ranged from undetectable levels of artemisinin in one product (Product A) to concentrations substantially exceeding the label amount in the other three (Products B, C, and D). The observed variability is not attributed to the HPLC technique because we used a USP analytical reference source as our standard, and the same method was applied to all samples. Moreover, the consistent reproducibility of the reference standard across multiple assay runs and various dilution factors reinforced the reliability of the HPLC technique.

We cannot determine the source of the variability or failure to meet our acceptance criterion for these capsules without additional undue study. Because these products are not regulated by the FDA, there is no assurance of the supplement strength, purity, or quality. Other investigators have also observed wide variability in strength among commercially available dietary supplements (Funk et al. 2018; Gershwin et al. 2010; Erland and Saxena 2017; Andrews et al. 2017).

The discrepancy we observed between labeled and actual strength of these capsules reinforces the necessity of analyzing unregulated supplements before incorporation into treatment protocols to ensure that the patients are receiving the proper dose intended. Additionally, Products B, C, and D had greater than 150% strength when compared to the reference standard, showing the need for analysis to avoid potential adverse effects from an excessive dose. While artemisinin is generally well tolerated, potential dose‐dependent side effects can occur, including gastrointestinal, cardiovascular, and neurologic signs (Shibeshi et al. 2021).

There were some limitations in this study. We analyzed capsules from only one bottle (one lot) of each formulation. It is possible that a larger sample size would have produced different results. However, the results show that consumers of these unregulated products may be misled by the label claims of capsule strength. Also, this study only evaluated product strength. Stability testing and identification of impurities was not performed.

## Author Contributions

**Alyssa R. Berman:** conception and design, data acquisition, data analysis and interpretation, manuscript writing. **Adam J. Birkenheuer:** conception and design, data interpretation, manuscript writing. **Emily L. Sorah:** conception and design, manuscript writing. **Mark G. Papich:** conception and design, data acquisition, data analysis and interpretation, manuscript writing.

## Ethics Statement

No animals were used in this study. This study evaluated an unapproved supplement used in animals.

## Conflicts of Interest

The authors declare no conflicts of interest.

## Data Availability

The data that support the findings of this study are available from the corresponding author upon reasonable request.
